# Analysis of risk factors for long-term mortality in patients with stage II and III tuberculous meningitis

**DOI:** 10.1186/s12879-024-09561-0

**Published:** 2024-07-01

**Authors:** Ling Wang, Zhihan Gu, Xiaoli Chen, Xiaomin Yu, Xiandong Meng

**Affiliations:** 1 Department of Emergency Medicine, West China Hospital, Sichuan University/ West China School of Nursing, Sichuan University, Chengdu, China; 2 Disaster Medical Center, Sichuan University, Chengdu, China; 3 Nursing Key Laboratory of Sichuan Province, Chengdu, 610041 China; 4 Department of Emergency Medicine, Laboratory of Emergency Medicine, School of Medicine, West China Hospital, Sichuan University, Chengdu, China; 5 Mental Health Center, West China Hospital, Sichuan University/West China School of Nursing, Sichuan University, Chengdu, Sichuan 610041 China

**Keywords:** Tuberculous meningitis (TBM), BMRC, Mortality

## Abstract

**Objective:**

To investigate risk factors associated with long-term mortality in patients with stage II and III tuberculous meningitis (TBM).

**Methods:**

This retrospective analysis examined patients who were first diagnosed with stage II and III TBM at West China Hospital of Sichuan University between January 1, 2018 and October 1, 2019. Patients were followed via telephone and categorized into survival and mortality groups based on 4-year outcomes. Multivariate logistic regression identified independent risk factors for long-term mortality in stage II and III TBM.

**Results:**

In total, 178 patients were included, comprising 108 (60.7%) males and 36 (20.2%) non-survivors. Mean age was 36 ± 17 years. Compared to survivors, non-survivors demonstrated significantly higher age, heart rate, diastolic blood pressure, blood glucose, rates of headache, neurological deficits, cognitive dysfunction, impaired consciousness, hydrocephalus, and basal meningeal inflammation. This group also exhibited significantly lower Glasgow Coma Scale (GCS) scores, blood potassium, albumin, and cerebrospinal fluid chloride. Multivariate analysis revealed age (OR 1.042; 95% CI 1.015–1.070; *P* = 0.002), GCS score (OR 0.693; 95% CI 0.589–0.814; *P* < 0.001), neurological deficits (OR 5.204; 95% CI 2.056–13.174; *P* < 0.001), and hydrocephalus (OR 2.680; 95% CI 1.081–6.643; *P* = 0.033) as independent mortality risk factors. The ROC curve area under age was 0.613 (95% CI 0.506–0.720; *P* = 0.036) and 0.721 (95% CI 0.615–0.826; *P* < 0.001) under GCS score.

**Conclusion:**

Advanced age, reduced GCS scores, neurological deficits, and hydrocephalus were identified as independent risk factors for mortality in stage II and III TBM patients.

## Introduction

Tuberculosis (TB) represents a leading cause of infectious disease related deaths globally. TB mortality continues to rise, with untreated cases resulting in up to 50% fatalities [1]. Per the WHO, over 100,000 individuals develop tuberculous meningitis (TBM) annually [2]. As the most severe manifestation of TB, TBM confers substantial neurological morbidity and high mortality. Approximately 80,000 adults were estimated to die from TBM in 2019, with around 30% average mortality [3, 4]. Overall mortality risk reaches 23% by 3 months and 25% by 12 months post-diagnosis [5], with associated disability or death approaching 50% [6–9]. This confers tremendous personal and public health burden.

Due to ambiguous initial presentation and diagnostic challenges, most TBM patients fail to receive timely intervention [10, 11]. Moreover, many present at later stages of illness (stage II/III) [12, 13]. Disease severity per the British Medical Research Council (BMRC) stratification correlates with outcomes; higher classification indicates greater mortality risk [14]. A prior meta-analysis found ~ 70% of TBM patients to have stage II/III disease [5]. Another systematic review and meta-analysis found that patients with stage II (28.5%) and stage III (64.8%) tuberculous meningitis have a significantly higher risk of death than stage I (17.5%) [9], but clear factors for death have not been identified. Such advanced disease denotes critical status with generally poor prognosis despite treatment. While some analyses have evaluated prognostic variables in stage I-III TBM [15–17], few studies have exclusively focused on delineating long-term risk factors for mortality in stage II and III patients. As outcome heavily relies on accurate diagnosis and effective therapy [15], this study aimed to identify prognostic indicators in advanced TBM as early as possible. Timely targeted treatment and nursing care may improve outcomes. We examined risk factors for long-term mortality in stage II and III TBM.

## Information and methodology

### Study subjects

This retrospective study examined adult patients (≥ 18 years) who were first diagnosed with stage II or III TBM at West China Hospital of Sichuan University between January 1st, 2018 and October 1st, 2019. Patients were included in the study according to the standard TBM case definition proposed by Marais, which included clinical criteria, cerebrospinal fluid criteria, brain imaging criteria, evidence of tuberculosis elsewhere, and excluded other diagnoses. Obtain: Alternative diagnoses must be confirmed microbiologically, serologically, or histopathologically [18].TBM severity was stratified based on the 2010 International Consensus [18] and British Medical Research Council (BMRC) criteria (grade I (GCS 15; no focal neurological signs), grade II (GCS 11–14, or 15 with focal neurological signs), and grade III (GCS ≤ 10)) [19]. Exclusion criteria comprised insufficient clinical data, incomplete lab or imaging tests, pregnancy/lactation, prior neuropsychiatric disorders, sequelae of stroke/brain tumors/trauma, and malignant tumors.

### Research method

Data was extracted from the hospital electronic medical record system. We collected the following data from patients at the time of admission. Collected information included demographics (age, sex, extraskeletal TB), vital signs, symptoms (fever, night sweats, cough, headache, meningeal irritation, neck stiffness, neurological deficits, cognitive impairment, altered consciousness, cranial nerve palsies, weight loss, nausea/vomiting, Glasgow Coma Scale [GCS] score), lab tests (platelet count, electrolytes, blood glucose, albumin, cerebrospinal fluid analysis), and brain imaging findings (hydrocephalus, infarction, basal enhancement/exudates, meningeal inflammation).

### Management

Patients received two months of intensive treatment(isoniazid (10–20 mg/kg/day; up to 1200 mg/day), rifampicin (10–20 mg/kg/day; up to 600 mg/day), pyrazinamide (20–30 mg//kg/day; up to 1500 mg/day), ethambutol (15–20 mg/kg/day; up to 750 mg/day), and dexamethasone (0.4 mg/kg/day; up to 16 mg/day) and ten months of continuous treatment (isoniazid (10–20 mg/kg/day, up to 1200 mg/day), rifampicin (10–20 mg/kg/day, up to 600 mg/day).

### Follow-up and outcome indicators

Inpatients were followed via electronic medical records. Discharged patients were contacted by phone. Cases lost to follow-up after three unsuccessful contact attempts were excluded. The primary outcome was all-cause mortality within four years of TBM diagnosis.

### Statistical analysis

Statistical analyses were conducted using SPSS version 22.0(USA, IBM analytics). Normal continuous measurements are presented as means ± standard deviations. Groups were compared via independent samples t-tests, while count data were analyzed using χ2 tests. Variables with *p* < 0.05 on univariate testing were entered into a multivariate logistic regression model. Multivariate binary logistic regression identified factors independently predictive of 4-year mortality in stage II and III TBM. Results were expressed as odds ratios with 95% confidence intervals. Model adequacy was evaluated through Hosmer-Lemeshow testing. The significance level α was defined as 0.05. Receiver operating characteristic (ROC) curves assessed the predictive utility of age and GCS scores for long-term prognosis.

## Results

### Demographics and clinical features

In total, 178 eligible patients were analyzed, including 108 (60.7%) males and 36 (20.2%) non-survivors. Mean age was 36 ± 17 years. Stage III patients showed markedly higher long-term mortality versus stage II (59.3% vs. 13.2%, *p* < 0.001). Compared to survivors, non-survivors demonstrated significantly older age (*p* = 0.014); faster admission heart rate (*p* = 0.046) and diastolic blood pressure (*p* = 0.046); and higher rates of headache (*p* = 0.004), neurological deficits (*p* < 0.001), cognitive dysfunction (*p* = 0.021), and impaired consciousness (*p* = 0.004). Non-survivors also exhibited lower GCS scores (*p* < 0.001). No other univariate differences reached statistical significance (*p* > 0.05) (Tables 1 and 2).

**Table 1:** General analysis of patients in the survival and mortality with stage II and III TBM^1^

Variable	Survival (*n* = 142)	Mortality (*n* = 36)	*P*
Male, n,(%)	90 (63.4%)	18 (50%)	0.142
Age, years, mean ± sd	35 ± 16	43 ± 20	0.014
Combined tuberculosis mycobacterial infection at other sites, n,%	15 (10.6%)	7 (19.4%)	0.148
clinical stage			< 0.001
Stage II	131 (86.8%)	20 (13.2%)	
Stage III	11 (40.7%)	16 (59.3%)	
admission vital signs			
Body temperature (°C), mean ± sd	37.09 ± 0.91	37.24 ± 0.88	0.352
Heart rate (beats/min), mean ± sd	83.37 ± 19.10	93.44 ± 18.06	0.046
Respiration (breaths/min), mean ± sd	20.45 ± 2.40	21.28 ± 3.38	0.093
SBP ^a^(mmHg), mean ± sd	123.61 ± 54.66	126.58 ± 21.28	0.750
DBP^b^(mmHg), mean ± sd	72.32 ± 15.30	80.03 ± 14.24	0.007

Note:^1^TBM: Tuberculosis meningitis;^a^SBP: Systolic blood pressure;^b^DBP: Diastolic blood pressure

**Table 2:** Analysis of clinical symptoms in patients with stage II and III TBM^1^ in the survival and mortality

Variable	Survival	Mortality	*P*
Fever (> 37.5℃)>5 days, n,(%)	120 (84.5%)	26 (72.2%)	0.086
Night sweats, n,(%)	37 (26.1%)	8 (22.2%)	0.636
Cough > 2 weeks, n,(%)	37 (26.1%)	9 (25.0%)	0.897
Headache, n,(%)	133 (93.7%)	28 (77.8%)	0.004
Neck stiffness, n,(%)	104 (73.2%)	29 (80.6%)	0.367
Peripheral nerve dysfunction, n,(%)	41 (28.9%)	23 (63.9%)	< 0.001
Cognitive dysfunction, n,(%)	29 (20.4%)	14 (38.9%)	0.021
Altered consciousness, n,(%)	77 (54.2%)	29 (80.6%)	0.004
Cranial nerve palsy, n,(%)	44 (31.0%)	11 (30.6%)	0.960
Weight loss, n,(%)	55 (39%)	11 (30.6%)	0.349
Vomiting, n,(%)	95 (66.9%)	19 (52.8%)	0.115
Nausea, n,(%)	82 (57.7%)	17 (47.2%)	0.256
GCS score	13 ± 2	11 ± 3	< 0.001

Note:^1^TBM: Tuberculosis meningitis

### Comparison of auxiliary examination results

Compared to survivors, non-survivors showed significantly higher blood glucose (*P* = 0.014), increased hydrocephalus (*P* < 0.001), and greater basal meningeal inflammation (*P* = 0.028). Conversely, non-survivors demonstrated significantly lower blood potassium (*P* = 0.009), serum albumin (*P* = 0.003), and cerebrospinal fluid chloride (*P* = 0.040). No other between-group differences in lab or imaging findings reached statistical significance (*P* > 0.05) (Table 3).

**Table 3:** Comparison of laboratory and radiologic features at admission between TBM stage II/III survival and mortality

Variable	Survival	Mortality	*p*
Laboratory data			
Platelets, 10^^9^ /L, mean ± sd	233.46 ± 89.45	221.25 ± 80.82	0.457
Blood potassium, mmol/L, mean ± sd	3.68 ± 0.48	3.44 ± 0.49	0.009
Blood leukocyte count, 10^^9^ /L, mean ± sd	7.92 ± 3.13	8.24 ± 4.35	0.611
Blood glucose, mmol/L, mean ± sd	6.07 ± 1.36	6.72 ± 1.61	0.014
Blood sodium, mmol/L, mean ± sd	131.70 ± 7.62	132.34 ± 6.78	0.646
Human blood albumin, g/L, mean ± sd	38.76 ± 4.71	35.89 ± 6.18	0.003
CSF^a^ blood glucose, mmol/L, mean ± sd	1.91 ± 0.97	2.16 ± 1.18	0.179
CSF^a^ protein, g/L, mean ± sd	3.08 ± 6.57	3.95 ± 4.77	0.453
CSF^a^ chloride, mmol/L, mean ± sd	113.74 ± 7.52	110.71 ± 9.08	0.040
Imaging			
Hydrocephalus, n,(%)	42 (29.6%)	22 (61.1%)	< 0.001
Cerebral infarction, n,(%)	60 (42.3%)	21 (58.3%)	0.084
Basement membrane exudate, n,(%)	8 (5.6%)	6 (16.7%)	0.028
Meningeal enhancement, n,(%)	42 (29.6%)	13 (36.1%)	0.449

Note:^1^TBM: Tuberculosis meningitis;^a^CSF: Cerebrospinal fluid

### Results of multifactorial analysis

A multivariate logistic regression analysis evaluated associations between candidate risk factors (age, admission vital signs, presenting symptoms, GCS, lab values, imaging findings) and 4-year mortality in TBM. Results identified advanced age (OR 1.042; 95% CI 1.015–1.070; *P* = 0.002), lower GCS scores (OR 0.693; 95% CI 0.589–0.814; *P* < 0.001), neurological deficits (OR 5.204; 95% CI 2.056–13.174; *P* < 0.001), and hydrocephalus (OR 2.680; 95% CI 1.081–6.643; *P* = 0.033) as significant independent predictors of long-term mortality (Table 4). The Hosmer-Lemeshow test produced χ2 = 7.391, *P* = 0.495, supporting adequate model fit.

**Table 4:** Analysis of independent factors for long-term mortality in stage II and III TBM^1^

Variable	*P*	OR^a^	95% CI^b^
Age	0.002	1.042	1.015, 1.070
GCS score	< 0.001	0.693	0.589,0.814
Peripheral nerve dysfunction	< 0.001	5.204	2.056,13.174
Hydrocephalus	0.033	2.680	1.081,6.643

Note:^1^TBM: Tuberculosis meningitis;^a^OR: Odds ratio;^b^CI: Confidence interval

### Predictive value of age and GCS score

**Fig. 1:**
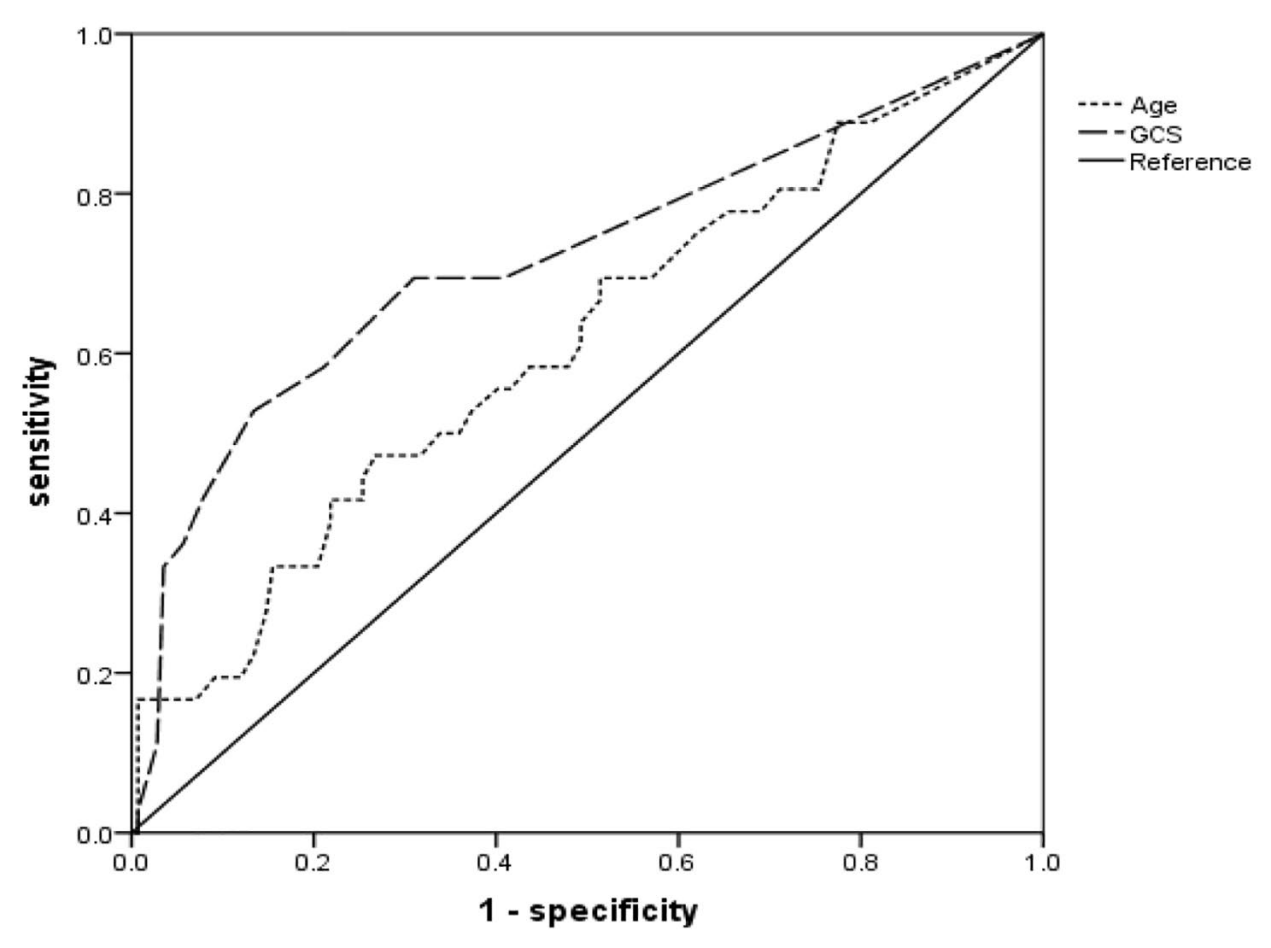
The ROC curves of age and GCS in predicting long-term prognosis in patients with Stage II and III TBM

ROC analysis examined the utility of age and admission GCS scores to prognosticate long-term mortality in stage II/III TBM(Fig. 1). For age, the area under the curve measured 0.613 (95% CI 0.506–0.720; *P* = 0.036), with an optimal cut-off of 44.5 years, 47.2% sensitivity, and 73.2% specificity. The GCS ROC area was 0.721 (95% CI 0.615–0.826; *P* < 0.001), with an optimal cut-point of 11, conferring 52.8% sensitivity and 86.6% specificity (Table 5).

**Table 5:** Predictive accuracy of age and GCS score for long-term mortality in stage II/III TBM patients

Variable	AUC	*p*	95% CI
Age	0.613	0.036	0.506, 0.720
GCS	0.721	< 0.001	0.615, 0.826

Note:^1^TBM: Tuberculosis meningitis

## Discussion

Previous studies have highlighted the association between the severity Tuberculous Meningitis (TBM) and negative clinical outcomes [20, 21]. Increased mortality rates have been linked to higher Medical Research Council (RC) classification scores [22], and more advanced stages of TBM have been associated with a six-fold increase in the risk of death in patients [15]. Our study reaffirms these findings, showing that the mortality rate in stage III patients is nearly four times higher than in those at stage II. Thus, it is critical to identify and monitor the factors influencing long-term mortality in patients with advanced TBM to improve their overall health.

In our study, we found that TBM predominantly affects younger adults, with the average age of stage II and III patients being approximately 36 ± 17 years. This is consistent with the findings of a previous study by Gu J [13]. The long-term mortality rate in our sample was 20.2%, which aligns with previous research [15, 23]. We observed that the age of patients in the deceased group was significantly higher than that of the survivors. Moreover, the majority of older patients were at stage II or III of the disease [24]. Gu J et al. [13] reported that 63.3% of TBM patients with poor prognoses were above 60 years of age. However, sample primarily consisted of young and middle-aged stage II patients, resulting in some inconsistencies. We identified a cut-off age of 44.5 (sensitivity 47.2%, specificity 73.2%, *P* = 0.036), suggesting that the risk of mortality increases with age. This could be attributed to the presence of multiple comorbidities in older individuals, weakened immune defense mechanisms, and suboptimal immune responses to mycobacterium infection [24].

In our study, the Glasgow Coma Scale (GCS) emerged as an independent predictor of long-term mortality in patients with Stage II and III TBM, corroborating previous studies that highlighted an association between low GCS scores and unfavorable outcomes [12, 13, 21, 25]. The strength of our study lies in its extended follow-up period, allowing us to gain a comprehensive understanding of the long-term survival prospects of TBM patients. The GCS is a widely used and reliable tool that assesses consciousness impairment in TBM patients by evaluating eye opening, verbal, and motor responses. It can also predict cognitive function and motor-related sequelae [26]. In our cohort, patients with lower GCS scores exhibited higher levels of consciousness impairment. We found that mortality rates significantly increased when GCS scores were11 (sensitivity 52.8%, specificity 86.6%, *P* < 0.001). Therefore, healthcare professionals should accurately assess and closely monitor changes in patients’ level of consciousness, pupil dilation, and other relevant indicators. Timely evaluation is essential for developing an individualized, evidence-based management plan.

Our multivariate analysis identified peripheral neurological dysfunction as an independent risk factor for long-term mortality in Stage II and III TBM patients. In TBM, mycobacterium tuberculosis invades the meninges and brain parenchyma, activating and penetrating the subarachnoid space with tubercle bacilli, which then disseminate with the cerebrospinal fluid (CSF). This can result in neurological dysfunction in various segments of the spinal cord [26]. Peripheral neurological deficits may manifest as hemiplegia, vision or hearing loss, ataxia, unresponsiveness, among others [27, 28]. In our study, 63.9% of long-term deceased patients presented with peripheral neurological symptoms, such as numbness, facial asymmetry, limb weakness, fine motor dysfunction, and urinary and fecal incontinence. These symptoms significantly impaired the patients’ self-care ability and negatively impacted their quality of life, leading to feelings of inferiority, guilt, and loneliness [29]. Quality of life and mental well-being significantly influence disease progression, and strong familial and social support are crucial in enhancing treatment adherence [30]. Therefore, it is essential to mobilize resources for patients diagnosed with advanced TBM to provide psychological support, alleviate negative emotions, and improve their motivation and confidence in adhering to their treatment regimen.

Existing literature suggests a strong association between hydrocephalus and adverse outcomes, including mortality, in TBM patients [31–33], particularly those with higher MRC classification. Our study reaffirms these findings. The inflammatory response elicited by mycobacterium tuberculosis infection in the subarachnoid space can lead to a viscous exudate obstructing the subarachnoid space at the brain base, causing hydrocephalus [21]. This common intracranial complication can occur at any stage of TBM and often results in increased intracranial pressure [34]. This may be a primary contributor to elevated intracranial pressure in TBM patients, leading to functional impairments affecting learning, memory, and movement [35], and in severe cases, coma, brain herniation, or death. Head CT/MRI is a reliable tool for diagnosing and assessing the severity of hydrocephalus in TBM patients [36, 37]. Medical professionals should closely monitor imaging results, especially signs of hydrocephalus, in Stage II/III TBM patients. Prompt review of CT or MRI scans is crucial if changes in consciousness level or pupils are observed.

### Limitations

The present study has several limitations, including its retrospective design, single-site sampling, reliance on our hospital’s patient records, and relatively small sample size. Future investigations would benefit from a larger sample size and a multi-center, prospective study design.

## Conclusions

Age, GCS score, peripheral neurological dysfunction, and hydrocephalus are independent predictors of long-term mortality in advanced TBM patients. Therefore, healthcare professionals should pay close attention to these clinical manifestations, enhance assessment procedures, and provide timely intervention.

## Data Availability

All data generated or analyzed during this study are included in this article.

## Abbreviations

BMRC: British Medical Research Council
TB: Tuberculosis
TBM: Tuberculosis meningitis
GCS: Glasgow Coma Scale
SBP: Systolic blood pressure
DBP: Diastolic blood pressure
CSF: Cerebrospinal fluid
OR: Odds ratio
CI: Confidence interval
ROC: Receiver Operating Characteristic
